# Unraveling HBV Life Cycle by Fluorescent Microscopy Imaging

**DOI:** 10.3390/v18080898

**Published:** 2026-08-14

**Authors:** Vanessa Sarabia Vega, Virgile Rat, Florian Seigneuret, Sébastien Eymieux, Philippe Chouteau, Hugues de Rocquigny

**Affiliations:** 1 INSERM U1259 MAVIVHe, Université de Tours and CHRU de Tours, 10 boulevard Tonnellé-BP 3223, 37032 Tours Cedex 1, France; florian.seigneuret@univ-tours.fr (F.S.); sebastien.eymieux@univ-tours.fr (S.E.); philippe.chouteau@univ-tours.fr (P.C.); 2 Domaines Membranaires et Assemblage Viral (MDVA), Institut de Recherche en Infectiologie de Montpellier (IRIM), UMR 9004, CNRS, 1919 Route de Mende, 34293 Montpellier, France; 3 Plate-Forme IBiSA des Microscopies, PPF ASB, Université de Tours and CHRU de Tours, 10 boulevard Tonnellé-BP 3223, 37032 Tours Cedex 1, France

**Keywords:** HBV, fluorescence, traffic, microscopy

## Abstract

The hepatitis B virus (HBV) is a worldwide hepatotropic virus despite the availability of a highly effective vaccine. Currently, the most efficacious treatment against HBV infection is the use of interferon and nucleos(t)ide analogs, which effectively suppress viral replication but do not eradicate infection. Consequently, studies are still ongoing to identify novel molecules with antiviral properties. Interfering with HBV trafficking in infected cells is an approach that would greatly benefit from the diverse techniques of microscopy. However, several challenges arise in employing such techniques for HBV, given the unique characteristics of this virus. Notably, HBV predominantly produces non-infectious subviral particles in addition to infectious virions. Moreover, both virions and SVPs are small objects, measuring only 20 to 40 nm, which challenges their observation using conventional optical microscopy. Additional obstacles include the small size of the HBV genome and its high compaction, which restrict reverse genetics and the introduction of DNA sequences coding for fluorescent polypeptides. In this review, we will provide an overview of ongoing research efforts aimed at visualizing the different stages of the HBV life cycle within infected cells. Furthermore, we will explore solutions successfully applied to the visualization of other viruses that could potentially be adapted to HBV.

## 1. Introduction

The hepatitis B virus (HBV) is the prototype of the Hepadnaviridae family. Hepadnaviruses are strictly hepatotropic and lead to transient or chronic infection in humans [1]. Worldwide, it is estimated that more than 250 million people are living with chronic HBV infection, placing them at increased risk of developing cirrhosis and hepatocellular carcinoma (HCC) [2].

**Figure 1:**
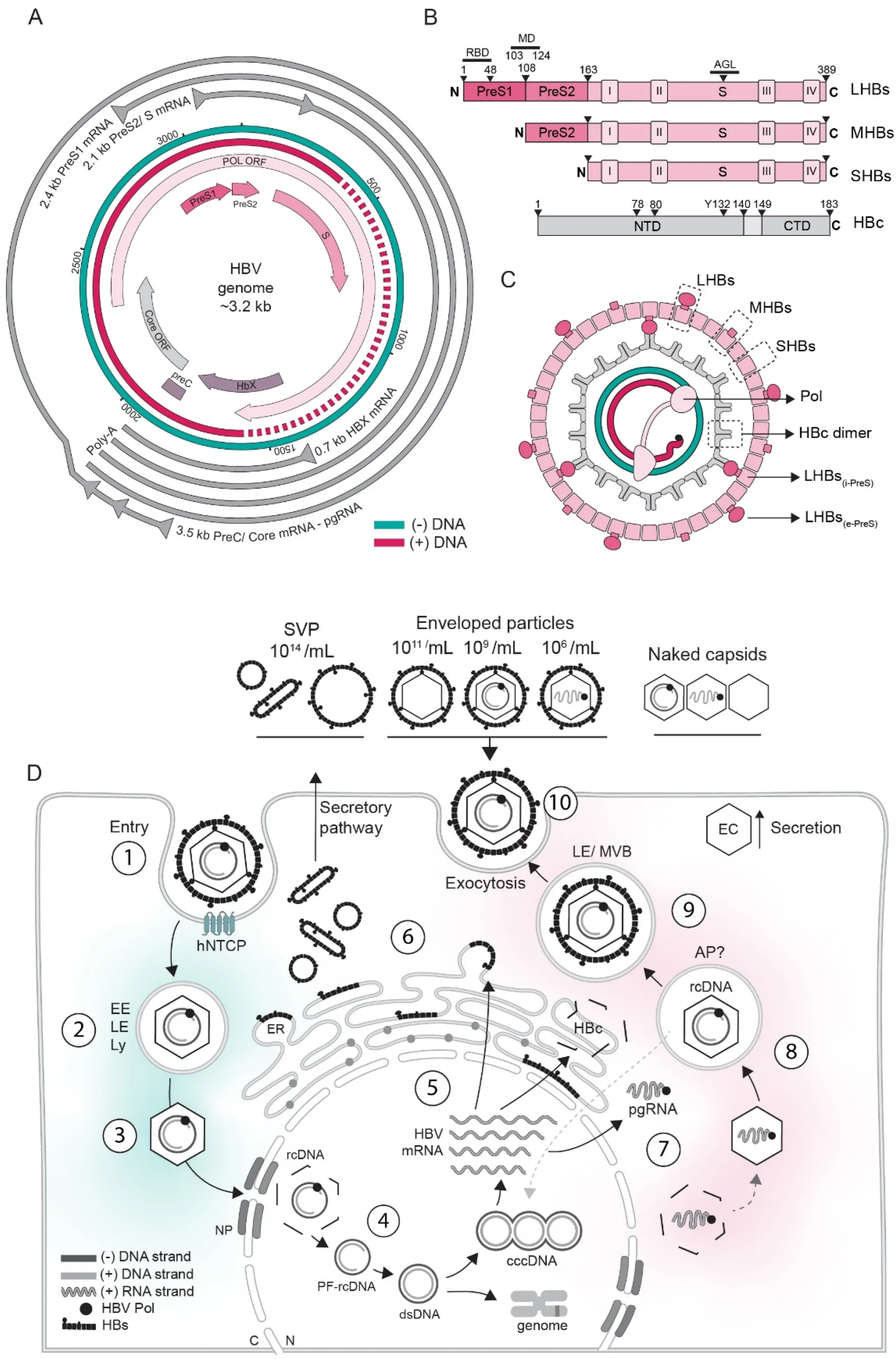
HBV genome, Dane particles, envelope proteins, and replication cycle. (**A**) Schematic representation of the 3.2 kb HBV genome with, from the outer to the inner circle, in gray, the mRNAs encoding the pgRNA/precore/core, the envelope proteins SHBs, MHBs, and LHBs, and HBx. In green, the rcDNA strand (−); in magenta, rcDNA (+); and, as colored circular arrows, the ORFs of the viral proteins. (**B**) HBV envelope proteins consist of LHBs, MHBs, and SHBs, sharing a common C-terminal domain corresponding to the 226 residues of the SHB protein. MHBs include an additional 55-residue preS2 domain, while LHBs include an additional 108–119-residue preS1 domain. A Matrix Domain (MD) mediates the interaction with the NC for virion assembly. In gray, HBc contains an N-terminal domain (NTD) essential for forming the icosahedral shell and a C-terminal domain (CTD) required for DNA replication. (**C**) The Dane particle is spherical, occasionally pleomorphic, and 42–50 nm in diameter, and is primarily composed of the mature nucleocapsid made of HBc (32 to 36 nm) (gray), the rcDNA (magenta and green), Pol (pink), and is surrounded by the envelope proteins (dark pink) and a lipid bilayer. (**D**) A brief summary of the HBV replication cycle starts with the virion attachment to the cell surface and hNTCP-mediated entry (1), followed by endosomal trafficking (EE, LE, Ly), acidic degradation of the envelope, and release of the NC (2). The NC is transported through microtubules to the NP (3). In the nucleus, NC disassembly and genome release give rise to cccDNA and linear DNA integration into the cell genome (4). HBV mRNA and pgRNA are translated from cccDNA and L, M, and SHBs, as well as and HBc proteins are produced (5). SVPs are secreted through the ER-Golgi secretory pathway (6). pgRNA is encapsidated (7) and reverse transcribed into rcDNA (8) in unknown compartments. The mature NC is transported possibly through the MVB and late endosome (LE) compartment, where envelopment occurs (9), followed by vesicle-mediated exocytosis (10). Empty (EC) and non-enveloped capsids can also be secreted by alternative mechanisms. The upper diagram depicts the variety and relative abundance of HBV particles that can be secreted.

The HBV virion, also referred to as the Dane particle, consists of an inner nucleocapsid (NC) composed of the core protein and containing a partially double-stranded circular DNA genome (rcDNA) (Figure 1A). The NC is surrounded by an envelope that includes three types of envelope proteins (SHBs, MHBs, and LHBs) (Figure 1B,C). These surface proteins mediate low-affinity attachment of the virus to heparan sulfate proteoglycan (HSPG) on the surface of human hepatocytes [3], followed by high-affinity binding of the LHBs, specifically the 2–48 preS1 region, to the bile acid transporter Na^+^-taurocholate co-transporting polypeptide (NTCP), with the involvement of the epidermal growth factor receptor (EGFR) at the basolateral membrane of hepatocytes (extensively reviewed in [4]) (Figure 1B,C). The NTCP structure, resolved at 3.3 Å using cryo-EM, reveals a wide hydrophobic tunnel formed by nine transmembrane helices linking the extracellular space to the cytoplasm [5,6]. The myristoylated preS1 domain of the LHBs e-PreS isoform interacts with N-terminal residues of NTCP (Figure 1C,D), forming a lasso-like structure that fits into this tunnel. The myristoyl group from preS1 is proposed to transfer from the viral envelope to the cellular membrane, where it anchors near NTCP. This interaction between preS1 and NTCP is blocked by a synthetic myristoylated preS1-derived peptide [7], also known as bulevirtide, thereby preventing HBV and HDV from entering the cells [8,9]. Currently, this peptide is used as a novel antiviral agent for the treatment of patients chronically infected with HDV [9].

After the interaction of LHBs with NTCP, the virion membrane fuses with the cell membrane, and it is internalized through the endosomal pathway (Rab5 and Rab7) to facilitate retrograde transport and the release of HBV DNA into the nucleus (Figure 1D) [10,11]. Recently, the scavenger receptor class F member 2 (SCARF2) protein was described as an endosomal receptor for the virus, with the luminal domain of SCARF2 interacting with the 89–90 region of LHBs, while its proline-rich cytoplasmic domain enables the delivery of the nucleocapsid near the nuclear pores [12]. In the nucleus, the rcDNA is first repaired to form covalently closed circular DNA (cccDNA) by host DNA repair machinery [13]. The cccDNA is transcribed into pregenomic (pgRNA) and subgenomic mRNAs by host RNA polymerase II. The pgRNA serves as the template for the translation of both HBV Pol and the core protein, and for reverse transcription [14]. HBV Pol binds to the packaging signal on the pgRNA, and both are encapsidated into the NC. Inside the NC, the pgRNA is reverse transcribed into rcDNA. Although envelope proteins (HBs) anchored in the ER membrane give rise to the secretion of SVPs through the ER-Golgi (ERGIC) secretory pathway [15], the budding and egress of virions depend on the endosomal sorting complex required for transport (ESCRT) and multivesicular body (MVB) pathway [16,17,18,19]. In addition, several autophagosome (AP) markers have been implicated in capsid assembly and egress, suggesting the involvement of the autophagic pathway in NC routing to the MVB or to budding sites [20,21,22,23,24]. Finally, naked capsids can bud independently of the ESCRT machinery, suggesting a non-vesicular exocytosis process mediated by ALIX. Notably, ALIX inhibition does not impair virion release, highlighting the existence of distinct export routes for enveloped virions and non-enveloped empty capsids [25]. The observation that different types of particles use different exit pathways correlates with the differences in the number of secreted DNA-containing virions, SVPs, and naked NC and capsids. This has been the main obstacle to determining the egress pathway of infectious virions.

**Figure 2:**
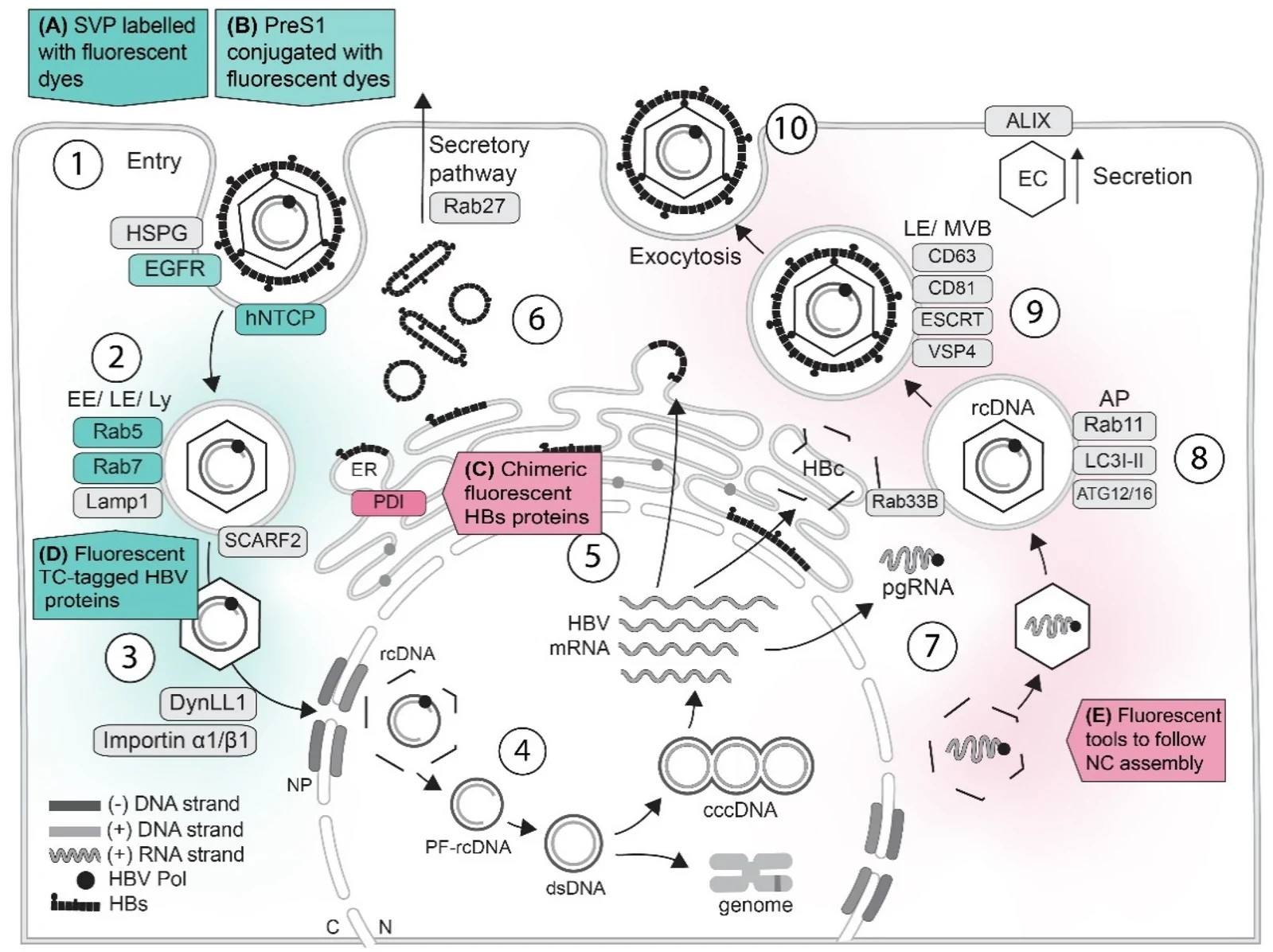
Insights into the HBV replication cycle revealed by fluorescence microscopy using tagged fluorescent proteins. Entry (green) and assembly-associated (pink) events that involve fluorescent modifications of HBV components are shown. (**A**) Labeled SVPs were developed to study viral entry. (**B**) Fluorescent-labeled PreS1 allowed the characterization of HBV-NTCP interaction determinants. (**C**) Chimeric fluorescent HBs proteins were used to track the cellular distribution of HBs. (**D**) The ReAsH/TC methodology has been used with HBc and LHBs to produce two labeled viruses, HepG2-NTCP, and to follow infection. (**E**) Fluorescently labeled HBc proteins were developed to follow capsid assembly. Several other cellular proteins associated with diverse HBV processes, identified by immunofluorescence microscopy, are also shown in gray.

Several steps of this viral cycle have been described due to major advances using viral proteins harboring a fluorescent tag (Figure 2). Some of these advances have already been reviewed [26,27]; here, however, we focus on more recent data obtained using quantitative fluorescence microscopy and on the use of fluorescence in situ hybridization (FISH) to image virion trafficking.

## 2. SVP Labeling with Fluorescent Dyes

Fluorescently labeled SVPs derived from HBV-infected human serum have been used as surrogates of virions for monitoring viral entry (Figure 2A). SVPs were purified and labeled with Bodipy (Bodipy-NHS). The attachment of Bodipy-labeled SVPs was assessed in HepG2 cells and primary cultures of human hepatocytes (PHH) [28]. It was observed by confocal microscopy that in PHH, the fluorescence accumulates in the cytoplasm in large, round vesicles, probably corresponding to internalized SVPs in lipid droplets [28]. In contrast, in HepG2 cells, which are not susceptible to infection because they lack NTCP expression, fluorescent bright spots remain at the surface of the cell.

A similar approach was further developed with commercial SVPs labeled with Cy5-NHS and the lipophilic dye DiI [29]. The labeled SVPs were then inoculated onto Cos7 cells. As a result, most of the fluorescence remained at the periphery of the cell. However, some intracellular bright spots were interpreted by the authors as evidence of a caveolin-dependent entry pathway. Although the labeling technique was technically valuable, this study had major limitations, as the non-hepatic Cos7 cells are non-permissive to HBV due to their lack of the NTCP receptor. In addition, there was no evidence that the commercial SVPs used contained the LHBs protein and, therefore, the PreS1 domain, which is critical for viral entry. These issues cast doubt on the physiological relevance of the findings.

An engineered HBV-HiBit-PS2 harboring the HiBit tag at the 5′ end of the PreS2 region has been assessed in HepG2-NTCP and PXB cells as a reporter system, demonstrating competence for replication and cccDNA production. The HiBit tag is an 11-amino acid luminescent peptide that reconstitutes the highly active NanoLuc luciferase upon high-affinity binding to its complementary LgBit subunit [30]. While both Dane particles and SVPs are produced similarly to those of wild-type HBV, the insertion of the HiBit tag into the preS2 region reduces the amount of cccDNA by a factor of 3 (in HepG2-NTCP cells) or by a factor of 10 (in PXB cells); therefore, the approach would be more appropriate for monitoring SVP trafficking and secretion. This indicates modifiable regions of the HBV genome where a split eGFP or TC could be inserted. However, there is no evidence of the intracellular effect of the HiBit tag on the HBV genome, nor of the production of intracellular rcDNA and pgRNA compared with those of the wild-type HBV. Further studies using larger fluorescent tags in the same region are needed to demonstrate that SVP trafficking is indeed not affected [31].

## 3. PreS1 Conjugated with Fluorescent Dyes

An alternative way of mimicking the first HBV-NTCP recognition step was to use a synthetic myristoylated preS1 peptide conjugated with Cy3 [7], FITC [32], Alexa594 [33], TAMRA [34,35,36], or Atto488 fluorescent molecules [37] (Figure 2B). The conjugated myr-preS1 peptide was essential for the identification of NTCP as the HBV receptor at the periphery of PHHs, differentiated HepaRG cells, and NTCP-expressing HepG2 or Huh7 cells. This tool was also helpful in discriminating between human NTCP (hNTCP) and non-human NTCP [32], defining the 157–165 NTCP domain as essential for preS1 binding and HDV/HBV infection [32,35], and identifying preS1 residues involved in NTCP binding [36]. Additionally, the use of this fluorescent tool facilitated the identification of feline NTCP [38] and many non-human NTCP orthologs [37]. Finally, conjugated preS1 peptides enabled the identification of compounds that inhibited the preS1–NTCP interaction and viral entry, including tauroursodeoxycholic acid (TUDCA), ezetimibe, and bile acids such as sodium taurocholate [33].

Fluorescent preS1 peptides were also used to track NTCP-mediated uptake into cytoplasmic vesicles [33] and to characterize the role of EGFR in the internalization of HBV [34] (Figure 2B). When HepG2-NTCP cells bearing TAMRA-preS1 were stimulated with EGF, preS1 and NTCP co-localized in large clusters in the cytoplasm, suggesting an interaction between the two receptors (NTCP and EGFR) [34]. More recently, when TAMRA-preS1 was combined with a proximity ligation assay (PLA) approach, it was shown that EGFR promotes the oligomerization of NTCP, which in turn facilitates the internalization of HBV from the cell surface to the endosomal network [39].

Interestingly, Murayama et al. were able to insert the HiBit tag notably at the N-terminus of the PreS1 region, overcoming the overlap with the Pol coding sequence, and to produce a HiBit-tagged HBV that can reproduce the entire viral life cycle in HepG2/NTPC cells. Like the HiBit introduction into the PreS2 region, the HiBit signal can be monitored in cell culture. While differences in particle production are observed, the initial entry step can be assessed with this system or with further fluorescent modifications in the context of viral attachment and entry receptor discovery [40].

## 4. Chimeric Fluorescent HBs Proteins

In fixed cells, proteins of viral or cellular origin are detected by indirect immunofluorescence using highly specific primary antibodies, which are subsequently recognized by fluorophore-conjugated secondary antibodies. This approach enables the visualization of protein localization, abundance, and subcellular distribution with high spatial resolution. However, dynamic studies on live cells rely on real-time microscopy and require labeling of the virus indirectly using encapsidated fluorescent proteins (such as CyclA-red or Vpr-GFP for HIV), or directly by appending a fluorescent protein to the protein of interest (referred to as FPs). These genetically encoded fluorophores led to a significant advance in virus tracking. The best-known example is GFP, a sequence extracted from the jellyfish *Aequorea victoria* [41,42]. Numerous GFP derivatives, notably eGFP (enhanced GFP), have been developed and centralized in a database (https://www.fpbase.org/, accessed on 1 July 2026) to obtain a colorful FP palette with great variety in terms of both biochemical and spectral properties [43,44,45]. In addition to covering a wide range of colors, properties such as pH dependence [46,47], temperature sensitivity [48], brightness [49], and photoactivation/photoswitching [50] have allowed the implementation of super-resolution microscopy (SRM) and have considerably improved the monitoring of protein–protein interactions by fluorescence energy transfer–fluorescence lifetime imaging microscopy (FRET-FLIM) [51]. FPs have been used extensively in retroviruses (see reviews [52,53,54]), orthohantaviruses [55], adeno-associated viruses [56], and influenza viruses [57].

In the case of HBV, FPs have been used primarily to study nucleocapsid and envelope assembly. Initially, two chimeric SHB proteins with an N-terminal eGFP (eGFP-SHBs) or a C-terminal yellow fluorescent protein (YFP) (SHBs-YFP) were constructed [58]. When expressed in Cos-7 cells, both proteins were detected in the cytoplasm, suggesting that FP-labeled SVPs could form and be released into the lumen of the ER. However, only chimeric SVPs composed of both eGFP-SHBs and wild-type SHBs were secreted into the supernatant. Similarly, the authors showed that by co-transfecting cells with a plasmid encoding the wild-type HBV genome and an eGFP-SHBs plasmid, eGFP-labeled DNA-containing particles were detected, which were able to interact non-specifically with the HepG2 membrane [58].

The eGFP reporter was also appended to the N-terminus of the MHBs protein. The chimeric protein eGFP-MHBs shares properties with wild-type MHBs, such as its glycosylation pattern, self-oligomerization, and co-localization with protein disulfide isomerase (PDI) in the ER at the nuclear periphery [59,60] (Figure 2C). When co-expressed with the complete wild-type HBV envelope protein repertoire, eGFP-MHBs was efficiently incorporated into fluorescent SVPs without affecting particle production, as SVP levels remained comparable to those observed in the absence of eGFP-MHBs. The fluorescent protein eGFP-MHBs can therefore substitute for wild-type MHBs without affecting SVP production. Therefore, fusing eGFP to the N-terminus appears more permissive than fusing it to the C-terminus for HBV particle expression. However, it remains to be determined whether co-transfection with a plasmid encoding the entire HBV genome and a plasmid encoding these chimeric proteins can generate a fluorescent infectious virus that would be compatible with replication in permissive cells.

## 5. Fluorescent Tetracysteine-Tagged HBV Proteins

While GFP has been the gold standard for tracking proteins inside cells, successful assembly of particles can be hindered by its size. Alternative approaches have been developed to generate recombinant viruses with small tags suitable for live cell imaging, such as the tetracysteine tag (TC). TC consists of a 12-cysteine-residue-rich peptide that interacts with the membrane-permeable biarsenical fluorescein derivative 4,5-bis (1,3,2-dithiarsolan-2-yl) fluorescein (FlAsH-EDT2; FlAsH) or its red analog, 4,5-bis (1,3,2-dithiarsolan-2-yl) resorufin (ReAsH-EDT2; ReAsH) [61]. Addition of this tag to HIV Gag does not interfere with Gag localization or assembly at the plasma membrane, virus infectivity, packaging of the viral Vpr protein, or interaction with TSG-101 [62,63,64,65,66,67,68,69]. The TC sequence has also been introduced into the capsid proteins of Borna disease virus [70,71] and Zika virus to generate replication-competent virions for tracking viral entry and intracellular trafficking in Vero cells [72]. Similarly, introducing this tag into the nucleoprotein sequence of the influenza virus demonstrated the accumulation of influenza virus components around the microtubule-organizing center (MTOC), establishing the role of Rab11a-positive vesicles in its morphogenesis [73]. Although the FlAsH or ReAsH/TC methodology is highly attractive due to the small size of the tag, which is expected not to affect the functions of the protein of interest, its application is challenging due to the high fluorescence background and the cytotoxicity generated by free FlAsH/ReAsH molecules [69,70,74].

Regardless, this strategy has been used in HBV by inserting the TC into the NTD (at positions 49 and 79) and the CTD (at position 155, hereafter referred to as TC-155) of HBc to determine capsid microtubule-dependent motility (Figure 2D) [75]. The signal corresponding to the labeled HBc-TC-ReAsH co-localized with the signal obtained using anti-HBc antibodies, suggesting that ReAsH was efficiently captured by TC-tagged HBc [75]. More recently, the HBc TC-155 chimeric protein was successfully used together with an LHB labeled with TC in its pre-S1 domain. In an original way, the two viruses, ReAsH-core-TC155HBV and ReAsH-LHBs-preS1HBV, were produced in HepG2.2.15 cells following transduction with HBV-CAT-TC155 and HBVCAT-TC-PreS1, respectively, purified, and then labeled in vitro by the addition of ReAsH [76]. When HepG2-NTCP cells were inoculated with the labeled virions, the infection was monitored over time with an HM-1000 super-resolution microscope (~40 nm). Within the first 30 min post-inoculation with ReAsH-PreS1HBV, the TC-labeled HBsAg protein was localized in Rab5-positive cytoplasmic vesicles, whereas at 60 min post-inoculation with ReAsH-TC- 155-coreHBV, the HBcAg signal accumulated in Rab7-positive vesicles, the Golgi, and the nucleus. Through careful analysis of the time course co-localization between either HBsAg or HBcAg and eGFP-labeled markers of cellular compartments, Li et al. showed that the HBV virions lose their envelope early after endocytosis [76].

## 6. Fluorescent Tools to Follow Assembly

### 6.1. Co-Expression of HBc-eGFP and Wt HBc for Capsid Trafficking Examination

While the studies described above have primarily focused on live imaging of HBV attachment, entry, and post-endocytic disassembly, less information is available on the assembly kinetics of newly produced capsids and virions (Figure 2E).

HBc constructs carrying GFP or mCherry inserts at the tip of the HBc spike (positions 78–81) were shown to be permissive for capsid assembly in vitro [77,78,79]. Moreover, the functionality of a chimeric core protein with FPs inserted into the c/e1 epitope loop was recently examined by substituting eGFP with Neon Green (Ng) [80]. The authors showed that co-expression of HBc-Ng and wt-HBc at a 1:1 ratio had no impact on HBV ssDNA synthesis, suggesting that the pgRNA had been properly encapsidated and reverse transcribed. The use of this tool to trace HBc in live cells showed that after transfection, it was first located in the nucleus. The nucleus was proposed as a site of capsid assembly [81,82], consistent with recent data showing that pgRNA is encapsidated in the nucleus [83]. Additionally, it was observed that in live cells monitored over time (up to 72 h), capsids, but not the capsid-deficient mutant, accumulated in the cytoplasm concomitantly with cell division [80]. The images also showed that the assembly-competent core protein did not return to the nucleus during interphase, suggesting the presence of a retention signal in the cytoplasm. The authors therefore proposed that the capsids traffic from the nucleus to the cytoplasm concomitantly with nuclear membrane breakdown during cell division. This is in contrast to studies proposing that capsids are exported through the nuclear pores by the nuclear export signal (NES) in the HBc CTD or by CRM1-mediated NES clusters at the conformationally flexible spike tip regions of assembled HBc capsids [22,83,84]. However, it is unlikely that HBV is dependent on cell division for its replication, considering that hepatocytes are non-proliferative cells [85] in the non-carcinogenic liver, and that cccDNA appears unstable during mitosis [86]. It should also be noted that this work was carried out in the absence of envelope proteins, whose interactions with HBc could substantially influence its intracellular fate [87,88,89].

### 6.2. Identification of Core Domains Involved in HBV Assembly Using HBc-eGFP

**Figure 3:**
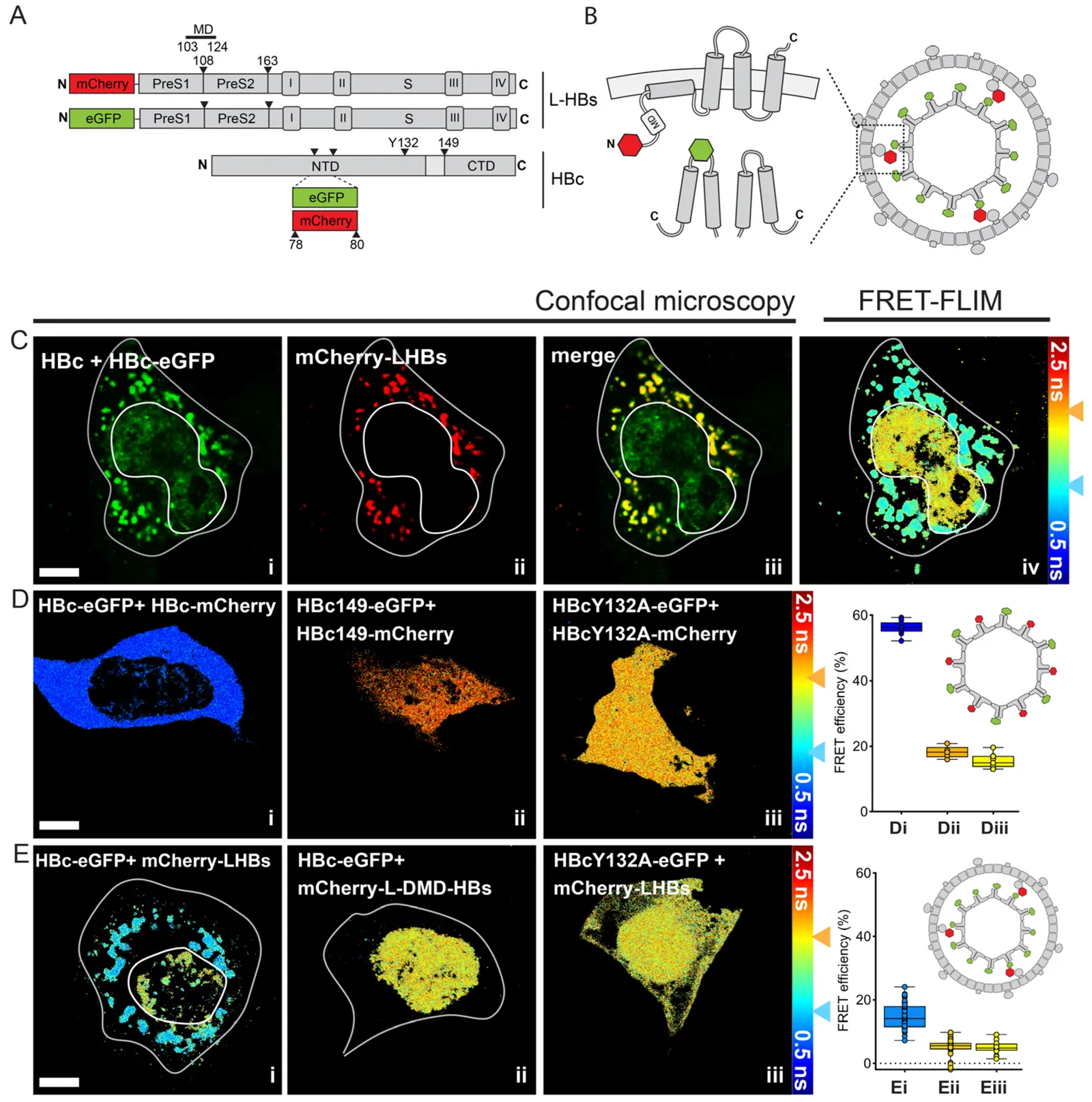
HBV assembly by FRET-FLIM. (**A**) The scheme represents a modified version of the LHBs and HBc proteins shown in Figure 1B. The black arrows between PreS1 and PreS2, and between PreS2 and S, indicate the AUG start codons, which were changed to ACG to prevent translation of the M and SHBs proteins. The mCherry and eGFP proteins were placed at the N-terminus of LHBs. The lower scheme shows HBc, in which eGFP or mCherry was inserted between positions 78 and 80 (middle black arrows). The Y132A mutant site is shown with a dark arrow. (**B**) (**Top**) The i-PreS topology of LHBs labeled with mCherry, and (**bottom**) the HBc dimer harboring eGFP at the top of helix 3. The scheme on the right shows the interaction of the MD of m-Cherry-LHBs with HBc-eGFP in the context of an assembled enveloped capsid. (**C**) Representative images of live cells expressing HBc-eGFP and mCherry-LHBs observed by confocal microscopy. The green channel (Panel i), the red channel (Panel ii), and the merged image (Panel iii) show confocal microscopy, whereas fluorescence lifetime measurements (FRET-FLIM) are represented in Panel iv. The bluish color in Panel iv clearly indicates that the HBc-LHBs interaction occurs in the cytoplasm at the periphery of the nucleus. (**D**) Live cells expressing HBc-eGFP together with HBc-mCherry were observed by FLIM. The navy blue color of the cell shows a high FRET efficiency corresponding to the tight self-oligomerization of HBc during capsid assembly (panel Di, navy blue). Panels Dii and Diii show lower FRET efficiencies of HBc149 (orange) and HBcY132A (yellow) mutants, respectively, indicating weak self-oligomerization. (**E**) The same representative experiment as in C: cells expressing HBc-eGFP and mCherry-LHBs (Ei), HBc-eGFP and mCherry-L-∆MD-HBs (Panel Eii), or HBcY132A-eGFP with mCherry-LHBs (Panel Eiii). The yellowish color of Eii et Eiii indicates the absence of interaction between the mutants in the context of the HBC-LHBs interaction. On the right, an arbitrary color scale ranges from blue (short lifetime, i.e., FRET) to red (long lifetime, i.e., absence of FRET). The box-and-whisker plots on the right of Panels D and E show the percentage of FRET efficiency. The colors of the boxes and dots correspond to the images (i, ii, iii) and the arbitrary color scale. The white scale bar represents 10 µm. White lines delineate the cell nucleus and cell membrane.

The self-assembly of HBc into an icosahedral capsid was well documented in vitro [90]. HBc is schematically divided into two functional domains, the NTD (residues 1–140) and the CTD (residues 150–183), united by the linker sequence (residues 141–149, 141-STLPETTVV-149) (Figure 1B and Figure 3A). In vitro, numerous studies have shown that the NTD is necessary and sufficient to form a capsid when produced at high concentration in a defined buffer. However, the sole expression of the NTD in permissive cells, or in rabbit reticulocyte lysate (RRL), does not allow capsid formation [91,92]. The crucial role of the CTD in capsid assembly was confirmed in our lab by combining quantitative fluorescence microscopy techniques, such as fluorescence lifetime imaging microscopy (FLIM) with Förster resonance energy transfer (FRET) (Figure 3) and fluorescence correlation spectroscopy (FCS to dissect assembly-related molecular interactions. In addition, we used transmission electron microscopy (TEM), a powerful method for visualizing viral and cellular structures at sub-nanometer resolution [81]. In FLIM, eGFP serves as the energy donor and mCherry as the acceptor, enabling FRET only when they are less than 5–7 nm apart—a range compatible with direct protein–protein interaction [93]. FLIM measures the donor’s fluorescence lifetime (τ) at each pixel, and FRET causes a reduction in τ, thereby allowing false color FLIM images to map the spatial distribution of HBc oligomerization (Figure 3D). FCS measures fluorescence fluctuations of HBc-eGFP within the femtoliter-scale focal volume, reflecting the translational dynamics of fluorescent molecules. When performed in live cells, FCS provides physical parameters such as diffusion time, local concentration, and molecular brightness; the latter indicating the average photon emission rate per particle, enabling stoichiometry analysis of oligomers [94].

By using fluorescently labeled HBc, we found that 80% of the HBc diffused as an oligomeric form [81]. This is in agreement with previous reports showing that HBc produced in hepatocytes is predominantly found as assembled capsids rather than free proteins [95]. We concluded that a concentration of 0.7–0.8 μM of HBc is sufficient to form capsids. This concentration is far below the concentration of NTD needed to form capsids in vitro [96], and it agrees with findings from experiments conducted in Xenopus oocytes [97]. This quantitative fluorescence microscopy approach was combined with sub-nanometer resolution TEM to verify that fluorescent oligomers corresponded to native capsids [98]. Additionally, by introducing deletions into the wt HBc protein, or its fluorescent derivatives, we showed that the NTD retains the property of forming small oligomers but, in contrast to the in vitro results, these oligomers never assemble into capsids. This confirms that the CTD facilitates the formation of capsids via its binding to cellular or viral RNA and through a protein–protein interaction mechanism [92,99,100,101,102].

More recently, the same technical approaches were used to investigate where the interaction between HBc and LHB proteins occurs [103] (Figure 3B,C). In this work, eGFP was placed at the tip of the HBc spike, while mCherry was positioned at the N-terminus of LHBs. The localization of the reporter was dictated by previous work showing that the addition of a protein or peptide at the N-terminus of LHBs had little impact on the protein’s function [40,58,59,60]. By using FLIM and TEM, we showed that HBc must assemble into capsids prior to its recruitment by LHBs to the nuclear periphery (Figure 3E). Notably, the capsid-LHB interaction depends on the matrix domain of LHBs but not on the SHB domain. Interestingly, while capsids are clearly distributed throughout the cell, including the nucleus, in the absence of LHBs, they condense and form clusters at the ER and late endosomal membranes in the presence of LHBs.

## 7. Fluorescent Labeling Techniques to Image HBV RNA and DNA

**Figure 4:**
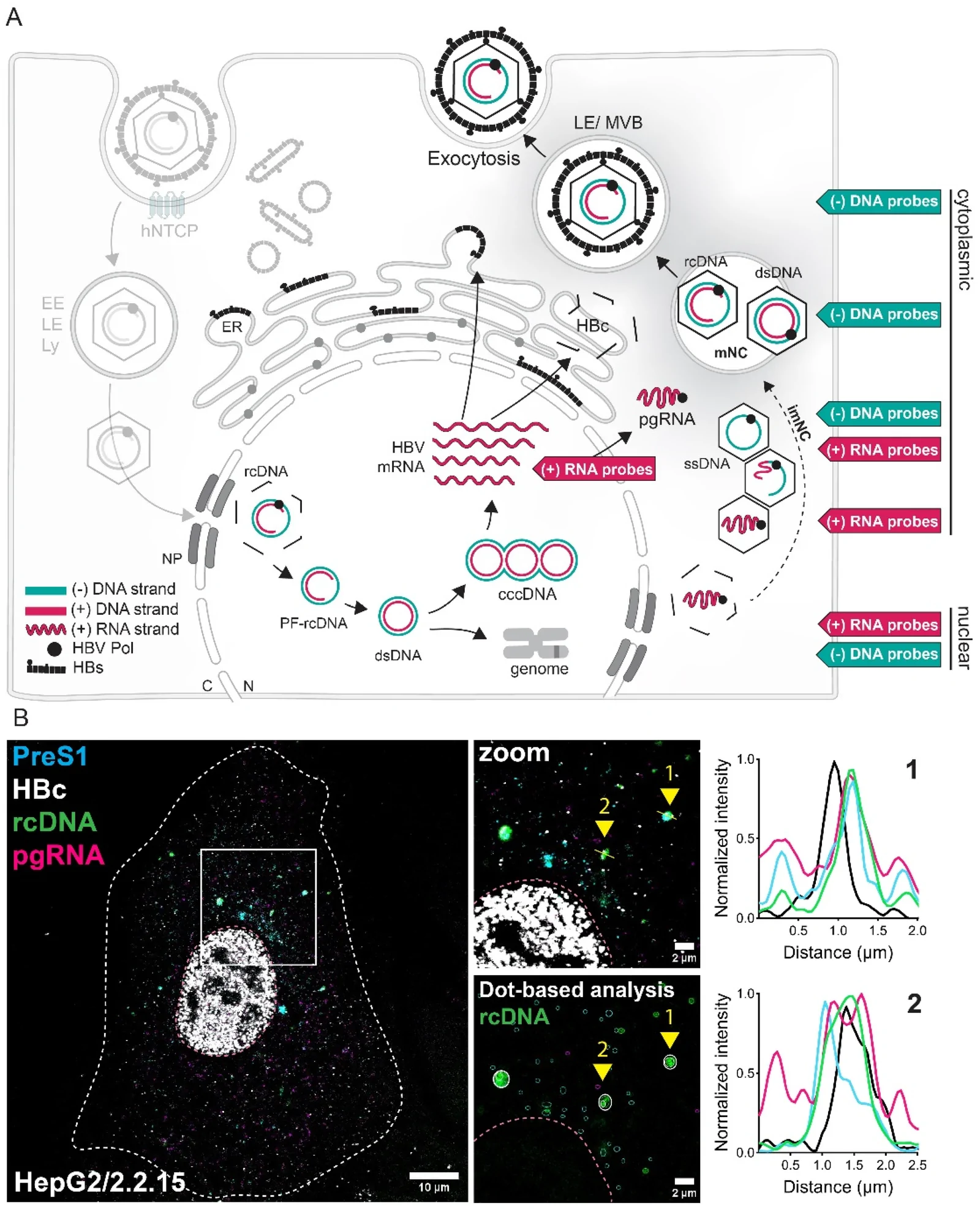
The nucleic acid soup inside HBV-infected cells. (**A**) The figure shows the variety of nucleic acid-containing intracellular particles that can be found in HBV replication-competent cell lines. On the right, probes generated against the (+) DNA strand (magenta) will detect pgRNA but will not be able to exclude nuclear HBV mRNA (curved magenta lines). While probes against the (−) DNA strand (green) will target nuclear and cytoplasmic HBV DNA species but will not differentiate between their intermediate forms. (**B**) Confocal microscopy images of HepG2/2.2.15 cells labeled by IF-smFISH against the (−) DNA strand. Probes directed against rcDNA (green) and HBV whole-genome probes directed against the pgRNA (magenta) were used in combination with antibodies against PreS1 (cyan) and HBc (gray). The top magnified (zoom) image shows dots 1 and 2 (indicated by yellow triangles) as areas in which pixels from all four channels co-localize in low HBV-expressing cells. The dotted fluorescence pattern of the signals can be assessed by dot-based analysis (bottom). Histograms show the intensity of the fluorescent signals (rcDNA—green, pgRNA—magenta, PreS1—cyan, HBc—black).

The HBV replication cycle includes the production of SVPs in amounts far exceeding those of DNA-containing virions (Figure 1D Top). Within the intracellular milieu, all particles—SVPs, empty particles, and DNA-RNA-containing particles, including those sorted for secretion and those sorted for degradation—are present together, creating a highly variable array of nucleic acids (NAs) coming from HBV infection (Figure 4A). Upon viral entry, NCs are imported into the nucleus, where rcDNA is deproteinized, generating the protein-free rcDNA (pf-rcDNA) that is repaired into double-stranded DNA (dsDNA) and then covalently closed circular DNA (cccDNA). The latter can persist in an episomal form, but dsDNA can also be found integrated into the host genome. The cellular transcription machinery produces viral mRNA from cccDNA, including pgRNA, which is encapsidated with HBV Pol as a ribonucleoprotein complex, forming short-lived immature particles.

Subsequently, the encapsidated pgRNA is reverse transcribed into rcDNA, resulting in mature DNA-containing NCs and, hence, mature DNA-containing virions after envelopment. Although the latter describes a straightforward process, capsids and virions containing NA intermediates, intermediates between pgRNA, ssDNA, and rcDNA, can eventually be secreted, and their transient presence in the cytoplasm has not been described [104]. At least, non-enveloped DNA-/RNA-containing NCs, referred to as naked (nucleo)capsids, were found to be released from infected cells [25].

While biochemical identification of these intermediates has been achieved, differentiating these species in a single-cell context has proven to be beyond the technical limits of DNA/RNA labeling and microscopy resolution. Indeed, as shown in Figure 4A, while probes against the (−) DNA strand could detect rcDNA and cccDNA, they cannot exclude the detection of nuclear pf-rcDNA and dsDNA transient intermediates (Figure 4A). In the same way, probes generated against the (+) RNA or (+) DNA strand can detect pgRNA under stringent conditions but cannot exclude preCore mRNA or other mRNAs.

Thus, the diverse array of transient and stable NA species of HBV makes it difficult to identify targets of interest and could explain why very few reports have included the detection of HBV NAs for specific tracking of NCs and virions. Besides the overlapping nature of the HBV genome, FISH has been demonstrated to be a powerful tool for identifying and localizing different forms of HBV NAs throughout the viral life cycle and for filling the gap in the sorting mechanism between empty and DNA-/RNA-containing particles (Figure 4B).

### 7.1. FISH Principles

**Figure 5:**
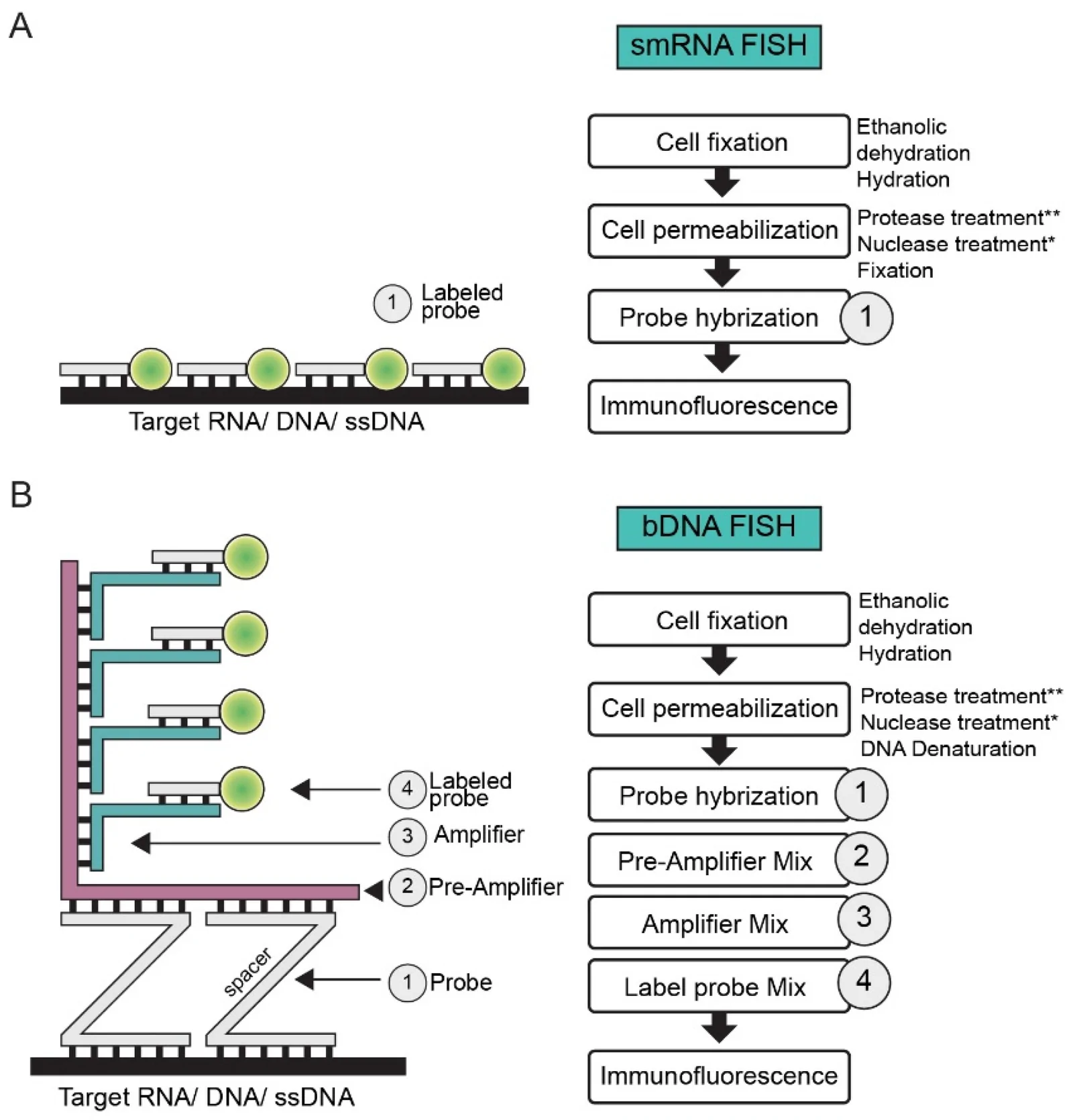
Differences and similarities between smFISH and bDNA FISH. Common steps between smFISH (**A**) and bDNA FISH (**B**) include cell fixation with paraformaldehyde (PFA). The bDNA FISH commercial protocol suggests detergent-mediated permeabilization, after an ethanolic gradient, dehydration for sample storage, and rehydration, while the smFISH commercial protocol recommends ethanolic permeabilization. Cell permeabilization using detergents is also recommended in the case of simultaneous or sequential immunofluorescence for smFISH. Probe hybridization can occur directly after ethanolic permeabilization in smFISH commercial protocols, but in the case of commercial bDNA FISH protocols, protease (**) and nuclease treatments (*) prior to labeling have been reported. There is no information about the same modification of the protocol in the commercial version of smFISH. In smFISH, the ssDNA probes specifically target RNA at 37 °C, and no denaturation step will favor DNA binding instead of RNA binding, while bDNA FISH commercial protocols recommend using a DNA denaturation step. The hybridization step is the main difference between the two protocols and determines the experimental complexity. smFISH has one probe hybridization step (1), while bDNA FISH has four (1–4). For IF-smFISH, the smFISH commercial protocol recommends doing IF first, and then smFISH; however, simultaneous co-labeling is also suggested. The bDNA FISH protocol suggests doing IF after FISH.

While it is somewhat simple to track the intracellular trafficking of one or more proteins at the same time by immunostaining or by co-expression of fluorescent derivatives, it is more delicate to visualize NAs through FISH. This technique is based on the complementary pairing between a designed probe and the intracellular DNA or RNA target [105] (Figure 4B and Figure 5). Intracellular NAs are fixed prior to hybridization with an oligonucleotide probe labeled with a fluorescent molecule. Alternatively, oligonucleotide probes can be peptide NAs (PNA), locked nucleic acid (LNA) analogs [106,107], or biotinylated and detected by fluorescent streptavidin or a fluorescent anti-biotin antibody (for recent reviews, [27,108]). Independently of the type of probe or FISH technique, intracellular HBV NAs are observed as a few dispersed dim or bright dots depending on whether confocal or high-resolution microscopy is used, respectively (Figure 4B). Probe specificity, sample pre-treatment techniques, image analysis, and microscopy power play a substantial role in interpreting results. Although a limited number of FISH techniques have been used in the HBV field, protocols may vary significantly from one study to another. A major source of variability arises from the protocols employed for nucleic acid deproteinization prior to labeling, an issue that becomes particularly critical given that the target HBV NAs are protected within the viral capsid. The protease digestion protocols recommended are not precisely described; they usually follow cell fixation and permeabilization, and the effects on capsid permeabilization, protein/NA disruption, or probe accessibility to the targets have not been reported. In addition, treatment with nucleases (DNAse or RNAse) to further distinguish between NA species might not be efficient when NAs are encapsulated in nuclease-resistant membranous vesicles. Below, we summarize the two main types of FISH methods used to detect different types of HBV NAs in infected cells (Table 1). Other protocols were described by Bustamante-Jaramillo et al. in 2022 [27].

### 7.2. RNA Single-Molecule FISH (smFISH)

To increase the sensitivity of FISH and reduce the signal-to-noise ratio, Tyagi’s group proposed probing the sequence of interest with a series of fluorescent DNA oligonucleotide probes containing 80–100 bases [120]. Depending on the size of the target sequence, probe sets (PSs) could include 25–50 fluorescent oligonucleotides. In this FISH approach, named single-molecule FISH (smRNA FISH), the overlap of these fluorescent probes increases the probability of hybridization and the size of the resulting bright spot [120] (Figure 5A). This technique has been used to track the RNA genome of several viruses, such as (i) the visualization of the initial steps of infection with the Rift Valley fever virus (RVFV) and its subsequent amplification throughout the cytoplasm [121], (ii) the tracking of HIV gRNA after reactivation of the latent HIV reservoir [122], and (iii) the detection of SARS-CoV-2 gRNA associated with nsp3 in replication organelles [122].

### 7.3. FISH Using Branched DNA (bDNA)

Branched-DNA (bDNA) is based on DNA amplification technology to detect RNA. It significantly reduces background fluorescence from nonspecific binding and improves the signal-to-noise ratio [123], but the major advantage is that signal enhancement depends on the hybridization of a cascade of complementary oligonucleotides, which bind in a branched, tree-like way (Figure 5B). In principle, the first probes, called Z probes, contain 18–25 bases complementary to the target RNA, a spacer, and a 14-nucleotide-long tail (1). Two tails need to be in proximity to form a binding site complementary to a second, pre-amplifier oligonucleotide (2). Next, a third L-shaped amplifier oligonucleotide, with L-shaped tails complementary to the pre-amplifier sequence, binds (3). The unbound part of the amplifier binds to the fluorescent probe, creating a cascade-like amplification of the signal (4) [124]. These fluorescent oligonucleotides can be labeled with different fluorophores to identify several RNA or DNA intermediates simultaneously [125]. The bDNA approach has been used to track the NAs of many viruses, notably for the spatiotemporal regulation of HCV replication [126], the accumulation of HCV RNA in exosomes [127], or the visualization of incoming HIV particles containing gRNA and/or nascent DNA [128].

While bDNA technology allows better amplification of the signal, it increases handling complexity and the possibility of contamination by nucleases. Nonetheless, the commercial version of bDNA FISH allows flexibility regarding the selection of the probes’ chromophore (Figure 5B–step 4) but has restrictions and dependence on the manufacturer regarding steps 1 to 3 (Figure 5B). Moreover, methodological adaptability of IF to FISH is critical for viral tracking. Nonetheless, in both cases, there is no information about how simultaneous or sequential IF affects the signal and how stable the fluorophore of the probe is during a standard IF protocol (37 °C incubation, BSA, Tween, etc.). Moreover, while bDNA FISH has two highly cited commercial versions on the market (RNAScope (Advanced Cell Diagnostics (ACD), a Bio-Techne brand, Newark, CA, USA) and ViewRNA ISH (Thermo Fisher Scientific, Waltham, MA, USA)), smFISH has only one (Stellaris (Biosearch Technologies, LGC, Petaluma, CA, USA)). Although bDNA FISH seems to have higher sensitivity than smFISH, it is important to note that smFISH can be modified in-house, allowing optimization under several co-labeling conditions. Therefore, it is recommended to take into consideration several factors before choosing a methodology, including the microscopy technique to be used (high-resolution, wide-field, or confocal microscopy), as the commercial versions of bDNA FISH and smFISH have been tested on different microscopes and have different arrays of fluorophores for labeling the probes; hence, they can be differently adapted to each user.

Regardless of the nucleic acid labeling technique, reproducibility relies on the posterior imaging modality employed, careful image processing, and quantitative analysis. The last two are essential to minimize background artifacts, segmentation bias, and false-positive colocalization. Additionally, appropriate nucleic acid and protein controls, which are not properly described beyond the use of nuclease treatment, need to be included and counted in each analysis. Standardized analysis workflows are therefore critical for the reliable interpretation of imaging data.

## 8. Detection of HBV NAs by FISH

### 8.1. Detection of HBV pgRNA in Infected and Producing Cells

**Figure 6:**
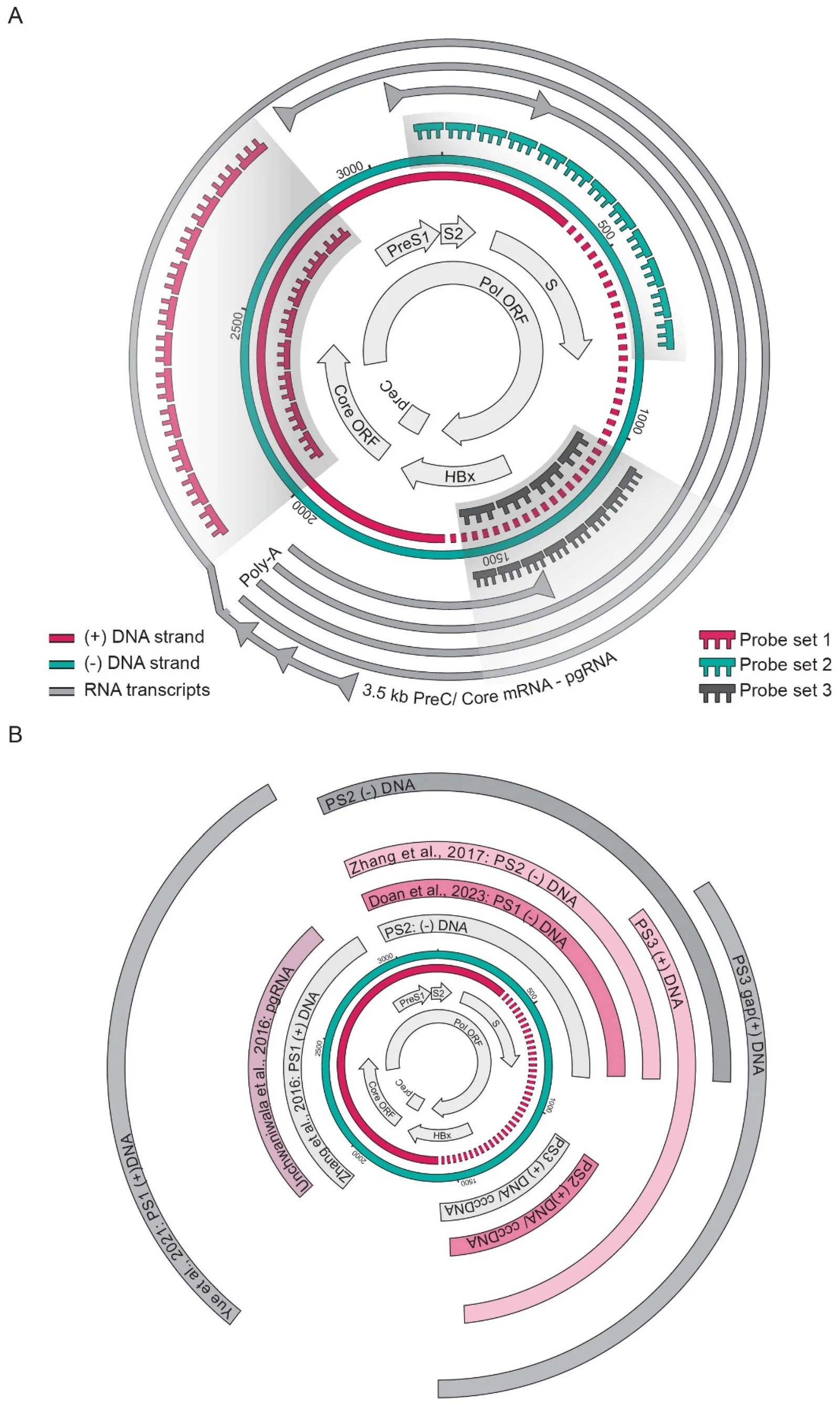
Detection of HBV nucleic acid species. (**A**) The (−) DNA strand and (+) DNA strand are represented by the green and magenta colors, respectively. The dotted magenta line corresponds to the (+) DNA gap region at its maximum expansion. This region is only present in cccDNA, dsDNA, and pf-rcDNA in the nucleus and is absent in rcDNA. The outer gray line represents the pgRNA (+) orientation, and the preCore/core mRNA, the inner gray lines represent the remaining subgenomic RNA transcripts. Probes complementary to the magenta (+) DNA strand are represented in the same magenta color. These probes can be used to identify HBV rcDNA and dsDNA in the cytoplasm, with cross-reactivity towards pgRNA and preCore/Core mRNA. Probes colored in gray, complementary to the dotted magenta (+) DNA strand, would identify cccDNA with cross-reactivity to dsDNA and dslDNA in the nucleus, as well as most RNA intermediates which have a (+) orientation. In this case, cellular localization and nuclease treatment are critical to define which NA species is being labeled. Probes complementary to the (−) DNA strand (green) can only identify DNA or DNA intermediates (and more specifically rcDNA if they were made complementary to the gap region). Dotted lines delimit the area of hybridization. (**B**) The hybridization region and strand orientation of each PS mentioned in Table 1, in the context of panel A. Arches of the same color represent the PSs used in the same publication but for different NA species (see Table 1) [109,110,112,113,118].

The smFISH protocol was used to specifically detect HBV pgRNA (3.5 kb pgRNA), Core and preC mRNA, but not subgenomic mRNAs (Pol, PreS1, S2, S) (Figure 1A). The PSs consisted of 26 singly labeled oligonucleotides specific to nucleotides 1987 to 2805 of the sequence of HBV genotype D (Figure 6A and referred to as PS 1 in Table 1) [109]. The experiment was carried out on HepG2-NTCP cells inoculated with 500 viral genome equivalents (VGE/cell) and observed by confocal microscopy seven days post-inoculation. HBV RNA was observed as 50 to 500 fluorescent dots per cell, homogeneously spread throughout the cytoplasm. The unimodal distribution of signal intensities suggested that each spot corresponded to a single pgRNA (or spliced derivatives) molecule. Even in the context of co-detection with HBc, it was not clear whether these dots could correspond to encapsidated or non-encapsidated pgRNA. The same type of fluorescent distribution was consistently observed in the cytoplasm of cells transfected with a replication-competent wt HBV DNA or an RT-deficient mutant.

Moreover, the presence of pgRNA was observed by bDNA FISH in the cytoplasm of HepAD38 cells and HepG2.2.15 cells (two cell lines that stably sustain HBV replication) using PS 1 (Figure 6), corresponding to PS 2 in Doan et al., 2023 [118] (see Table 1), and was confirmed by RNAse A/H treatment [110,111,113,118]. When fixed HepAD38 cells were indeed treated with RNAse A/H, the cytoplasmic signal was abrogated, and only a few residual bright dots were observed in the nucleus, possibly representing integrated HBV DNA or cccDNA depending on the stringency of the experimental settings [110,118]. As anticipated, the amounts of HBV pgRNA and DNA were greater in HepAD38 cells than in HepG2.2.15 cells and collapsed when HepAD38 cells were treated with doxycycline (i.e., under conditions of non-viral replication) [118].

The cytoplasmic distribution of pgRNA in cells derived from human liver biopsies was also documented using bDNA FISH with PS 1 (Table 1) after DNAse I treatment [26]. It should be noted here that these observations were made on histological sections of the human liver after colorization with NBT (Nitro blue tetrazolium chloride)/BCIP (5-Bromo-4-chloro-3-indolyl phosphate) staining, and counterstaining with nuclear fast red.

In addition to reporting the localization of HBV pgRNA or (+) DNA, FISH permitted the study of the translation of pgRNA. Indeed, the use of PS 1 combined with puromycin treatment allowed discrimination between: (i) free pgRNA, (ii) pgRNA undergoing translation, and (iii) pgRNA co-localized with HBc [110]. Based on the calculation of the distance between the pgRNA (FISH) and HBc (IF) carried out on HepAD38 or infected HepG2-NTCP cells in the presence of puromycin, the authors proposed that 3–4% of the pgRNAs were being translated, at a given time, and that 13.2% to 34.8% of pgRNAs co-localized with HBc. However, this co-localization could be limited due to the rapid diffusion of puromycilated peptides away from the translation site [129]. Interestingly, most of the pgRNA remained free (60.5 to 82.6%), and most of the capsids (64 to 90%) remained empty. We can also see from this work that the cellular translational machinery is poorly used, particularly in infected HepG2 cells, with only 2–3% of the ribosomes used, in agreement with the low level of viral mRNA [110]. To further corroborate these results, capsid assembly modulators (CAMs), BAY 41 4109 and GLS4, were used to induce large aggregates of core proteins that were segregated from pgRNA when cells were exposed to these compounds. Surprisingly, the size of the dots corresponding to active ribosomes increased, showing an aggregation of HBc as soon as it was produced. In the presence of CAMs, the co-localization between HBc and pgRNA decreased by a factor of two and, consequently, free pgRNA increased by two- to three-fold, confirming that CAMs disrupt the encapsidation of pgRNA by HBc aggregation.

### 8.2. Detection of Intracellular HBV DNA

PS 2 (Figure 6 and Table 1–referred to as PS 1 in Doan et al. [118]) was initially used to detect the (−) DNA strand. Hence, these probes would detect all forms of viral DNA, from pf-rcDNA to cccDNA [130]. The pf-rcDNA corresponds to an intermediate in the process of single-strand gap filling, deproteinization, nuclear transport, and cccDNA formation. The bDNA FISH with PS 2 allowed the observation of many bright dots present mainly in the cytoplasm of HepAD38 cells [110,111,118], as confirmed by high-resolution microscopy, STORM, and 3D-SIM [113]. These fluorescent signals were rarely observed after post-fixation DNAse I treatment. By contrast, RNAse-A/H treatment did not affect the signal, in agreement with the signals corresponding to DNA and not RNA species [110,118]. The use of PS 2 in HepAD38 cells, combined with immunostaining for preS1 and HBc, also demonstrated the scarcity of LHB and rcDNA co-localization, i.e., mature, DNA-containing virions [110,111]. Indeed, in both publications, an average of five rcDNA-associated dots per cell was observed in sharp contrast with the numerous fluorescent dots specific to LHBs or HBc. However, LHBs/rcDNA co-localization increased upon overexpression of LHBs, but not upon overexpression of MHBs or SHBs, consistent with the inhibitory effect of the LHBs protein on particle release [111]. A few residual nuclear fluorescent dots were observed after DNAse I treatment, possibly due to incomplete DNA digestion or to its protection from the enzyme in the NC [110].

To observe the kinetics of HBV NA accumulation, FISH was carried out on HepG2-NTCP cells after inoculation (MOI = 1000) with native or UV-irradiated HBV particles. Using PSs 1 and 2 [110], at one day post-inoculation (dpi), both HBV pgRNA and (−) DNA species were detected for both types of inoculum. However, despite a heterogeneous distribution of fluorescent dots from one cell to another, at 6 dpi, a few bright dots (~five puncta per cell) of HBV pgRNA and (−) DNA appeared solely in cells inoculated with virions not exposed to UV light, suggesting that signals observed by FISH were essentially from replication-competent viruses. Interestingly, the number of FISH signals per cell from pgRNA or (−) DNA plateaued at 15 days post-infection. Nevertheless, different kinetics of appearance were observed: HBV pgRNA increased linearly, while the amount of (−) DNA reached a steady state around 6 dpi.

Using the same infection approach on human hepatocytes isolated from PXB mice (PhoenixBio Co., Ltd., Hiroshima, Japan) (5 GEq/cell) and analyzing the cells by imaging and PCR [118], the different types of HBV NAs were detected as early as the first week using PSs 2 and 3. As expected, HBV RNAs and DNA were detected in the nucleus and the cytoplasm, while cccDNA remained localized in the nucleus. This contrasts with the results from Yue et al., where these NAs accumulated gradually as a function of time (21 days), and cells contained a greater number of fluorescent dots (about 15 dots/cell). In addition, the authors did not report differences in the kinetics of appearance between viral RNAs and (−) DNA, nor significant heterogeneity in the number of fluorescent dots from one cell to another [118].

The work carried out by Watashi’s group showed that HBc could interact with tubulin, and the authors proposed that such an interaction would favor capsid assembly [131]. By inducing tubulin inactivation with nocodazole, they showed an impairment of capsid assembly and a decrease in pgRNA packaging and viral DNA production. Therefore, the action of nocodazole on intracellular traffic would decrease HBV replication by directly affecting virion assembly. In fact, by FISH on HepAD38 cells using PSs 1 and 2, nocodazole (or vinblastine) treatment had a major effect on the cellular behavior of virions, since the blockage of microtubules doubled the number of mature particles (from ~5 to 10) at the periphery of the nuclei and revealed an inverse correlation between MVB size and viral (−) DNA puncta [110]. MVB size reduction was accompanied by an increased number of vesicles and enhanced viral (−) DNA colocalization with MVBs. These findings emphasize that microtubules participate in the fusion of smaller early endosomes during MVB formation, which in turn facilitates the transport of viral particles to the cellular periphery [17].

Another approach to track the (−) DNA strand involves using a series of probes covering the entire HBV genome [12,119]. For this purpose, 57 oligonucleotides, each consisting of 40 nucleotides, were selected based on their ability to hybridize to the HBV genome under the conditions used in the FISH method. These oligonucleotides were extended with 32 bases on both ends. These 32-base extensions were then targeted by additional oligonucleotides labeled at both the 3′ and 5′ ends with Alexa Fluor 647, allowing each of the 57 original oligonucleotides to be identified by four fluorophores.

Using this FISH methodology, Dingbin Tang et al. (2021) [119] demonstrated that in HepG2-NTCP cells infected with HBV-ΔX, the viral cccDNA preferentially localized near a transcriptionally inactive region of chromosome 19. Using FISH combined with the Oligopaints method [132], the authors showed that this region was positioned at the nucleolar periphery, a transcriptionally repressive nuclear compartment. In contrast, wild-type HBV cccDNA in HepG2-NTCP cells and primary human hepatocytes was enriched in transcriptionally active chromatin regions. Using a doxycycline-inducible HBx system, they found that HBV-ΔX cccDNA initially remained in inactive chromatin but relocated to active chromatin following HBx expression. Furthermore, depletion of Smc5/6 restored the localization of HBV-ΔX cccDNA to active chromatin, similar to wild-type HBV. These findings demonstrate that HBx promotes the redistribution of cccDNA from transcriptionally inactive to active chromatin by antagonizing the host restriction factor Smc5/6, thereby facilitating viral transcription.

Using the same FISH probes spanning the entire HBV genome, Wenhui Li and colleagues demonstrated that the host protein SCARF2 functions as an intracellular receptor that transports the HBV nucleocapsid from NTCP-containing endosomes to the nuclear periphery [12]. They identified the SCARF2-binding site within the preS1 domain at amino acids 89–90, distinct from the NTCP-binding region. Knockdown of SCARF2 reduced HBV infection in HepG2-NTCP, HepaRG, and primary human hepatocytes, whereas its overexpression enhanced viral infection. Deletion analyses further showed that both the luminal EGF4–6 domains and the cytoplasmic proline-rich domain of SCARF2 are required for its function. PLA revealed co-localization of SCARF2 and NTCP following infection, while FISH detected co-localization of SCARF2 with HBV DNA in the cytoplasm at 24 h post-infection. At later time points, HBV DNA was detected by FISH exclusively in the nucleus, and GFP-tagged SCARF2 was found to co-localize with both HBV DNA and the nuclear pore protein NUP153. Additional experiments confirmed that SCARF2 is associated with HBV in endosomes rather than lysosomes. Together, these findings support a model in which SCARF2 mediates nucleocapsid trafficking along microtubules to nuclear pores and may promote nucleocapsid release through liquid–liquid phase separation of its C-terminal domain.

### 8.3. Chromatinization of cccDNA

The rcDNA imported into the nucleus is repaired by the cellular machinery to produce cccDNA. The episomal cccDNA is essential for viral replication as a template for the cellular RNA polymerase II (Pol II) to produce the different HBV mRNAs. The cccDNA interacts with HBc, histones, and other epigenetic factors to form a mini-chromosome which is transcribed by Pol II [133]. As previously noted by Kann et al. [134], visualizing cccDNA by microscopy remains highly challenging due to its low copy number in the nucleus, the cross-reactivity of probes with replication intermediates (Figure 6), and the presence of integrated HBV DNA in infected cells.

Nevertheless, to monitor the fate of cccDNA through imaging, Seeger’s group used two cell models, Dstet5 and HepaD38, which express DHBV (Duck HBV) and HBV, respectively [115], and combined the Southern blot technique and FISH with biotinylated probes and avidin–fluorescein. These probes were incubated with purified cell nuclei to prevent the detection of cytoplasmic viral RNA or DNA. However, as they covered the entire genome of each virus, they could still detect integrated DNA, rcDNA, and double-stranded linear (dsl) DNA (Table 1). In the presence of doxycycline (i.e., viral replication off), two to four fluorescent dots were detected in both settings, due to leakiness of the doxycycline promoter, detection of integrated DNA, or non-specific recognition of cellular DNA. Conversely, in the absence of doxycycline (i.e., viral replication on), in cells cultured for 7 to 10 days (Dstet5) or three to four weeks (HepAD38), the number of fluorescent dots increased by a factor of five for DHBV and two to three for HBV. In these cells, the number of cccDNAs ranged from 0 to 60 copies for Dstet5 and 0 to 45 copies for HepAD38. This very broad distribution of cccDNA levels remains poorly explained. The authors suggested that it could be due to cellular factors guiding the nucleocapsid from the cytoplasm to the nucleus or to varying turnover rates of cccDNA between individual cells. Furthermore, the greater increase in cccDHBV DNA in comparison to cccHBV DNA was explained by the fact that Dstet5 cells contained env(−) DHBV DNA, thereby leading to an accumulation of NCs recycled to the nucleus, ultimately increasing the amount of cccDNA.

Additionally, they used the same approach to track cccDNA during cell division. Cells were treated with doxycycline (production of pgRNA from integrated sequences off) and then treated with tenofovir and analyzed by FISH and Southern blot at different time points. In both DHBV and HBV settings, the amount of viral DNA remained stable for the first few days, possibly because the rcDNA present at baseline matured into cccDNA. Then viral DNA slowly declined until day 17 for DHBV and day 25 for HBV, in accordance with cell division leading to a dilution of viral DNA in daughter cells [86].

More recently, PS 3, complementary to (+) DNA (initially reported by Zhang et al., in 2017 [113]), was generated [110,111]. In rcDNA, this (+) DNA strand sequence is absent, but present in cccDNA, dslDNA, and integrated HBV DNA (but under low-stringency binding conditions, it could also detect HBV RNAs). Using PS 3 and bDNA FISH (Figure 6 and Table 1), the authors built upon the work of Li et al. 2018 [115] and detected dots corresponding to cccDNA within the nucleus, with two to three times more signal in HepAD38 (dox-) cells than in HepAD38 (dox+) cells. They further used STimulated Emission Depletion (STED) (lateral resolution of ~30 nm) [110] and Super-Resolution Confocal Microscopy (resolution of ~50 nm) [118] to image cccDNA co-localized with euchromatin markers such as H3K27ac, H3K4me3 and RNA Pol II in HepAD38 and infected HepG2-NTCP cells, suggesting that the chromatinized cccDNA is localized in an active transcription region of the nucleus [110,118]. The cccDNA was also partially co-localized with markers of inactive chromatin, H3K27me3 and HP1a, in HepAD38 cells, suggesting that the transcriptional level of cccDNA may depend on cellular factors, the replication cycle, or cell division [118]. Furthermore, they depleted DOCK11 protein, involved in cytokinesis, and showed a decrease in cccDNA in active transcription areas (co-localization of cccDNA with both H3K4me3 and Pol II, markers of active transcription, decreased), suggesting that DOCK11, involved in cytokinesis, may regulate the subnuclear distribution of cccDNA or of other cellular factors [110].

Fluorescence microscopy also made it possible to study the movement of HBV and DHBV cccDNA, which is relatively stable in the nucleus, likely due to its interaction with proteins involved in repair and its anchoring to nuclear structures [135].

Since FISH is inaccurate for tracking cccDNA due to the need for cell fixation, a new method was developed using a CRISPR-Tag intron inserted into the HBV or DHBV genomes. This intron includes 18 dCas9 binding sites and is flanked by splice donor and acceptor sites, ensuring its absence in RNAs transcribed from cccDNA [114]. This recombinant cccDNA (rcccDNA-CRISPR-Tag for HBV and for DHBV)was produced in vitro and then transfected into HepG2 cells to demonstrate that the viral parameters were similar to those observed after transfection with pHBV1.3 or untagged rcccDNA. The authors also showed that this rcccDNA contained the expected epigenetic modifications on the cccDNA. In parallel, they developed a HepG2 cell line, referred to as DI3 cells, expressing dCas9-GFP11 (each dCas9 contains 14 peptides corresponding to GFP11) and GFP1-10-NLS [136]. When dCas9-GFP11 and GFP1-10-NLS interact, GFP is reconstituted in accordance with the split GFP principle, and a diffuse signal is imaged in the nucleus. When DI3 cells were co-transfected with the rcccDNA-CRISPR-Tag and a plasmid expressing the sgRNA (single guide RNA), the dCas9-GFP-NLS protein bound to rcccDNA-CRISPR-Tag, so that the images clearly showed a series of fluorescent dots corresponding to cccDNA in cells expressing HBsAg.

Interestingly, by performing FISH with probes targeting the intron of the rcccDNA-CRISPR-Tag, the dCas9-GFP and FISH probe signals co-localized, supporting the conclusion that the rcccDNA-CRISPR-Tag foci are located in actively transcribed regions. Notably, the deletion of the HBc protein from this construct had no effect on the number of cccDNA but, of course, it completely inhibited the formation of viral particles containing DNA. In cells transfected with an rcccDNA-CRISPR-Tag∆X plasmid (in which HBx expression was abrogated), an average of 2 foci per cell was detected instead of 5.5 foci in the presence of HBx. The authors proposed that the reduction in the number of foci was due to either the lack of the transcriptional activity of HBx or the impaired accessibility of the dCas9-GFP-NLS-sgRNA complex to the CRISPR-Tag sequence of the rcccDNA.

Importantly, cells containing the rcccDNA-CRISPR-Tag were observed during mitosis. As described in [86,115], the results confirm that cccDNA, in its episomal form, is evenly distributed among daughter cells and disappears during cell division.

Finally, the advantage of tracking a signal in live cells is the ability to observe cccDNA and its particularly slow movement (3.6 × 10^−3^ µm^2^/s and 3.8 × 10^−3^ µm^2^/s for HBV and DHBV CRISPR-tagged rcccDNA, respectively) over a small area of 0.2 µm^2^. This diffusion is much slower than GFP diffusion(90 µm^2^/s, [137]). However, there might be a bias in this result, as the rcccDNA contains 18 Cas9 target sequences, each with 14 GFP11 domains, potentially recruiting up to 14 GFPs. Therefore, the fluorescent object being tracked could be two to three times larger than the cccDNA alone. Nevertheless, the diffusion coefficient remains low, which supports the idea that the cccDNA may be retained by internal nuclear structures.

## 9. Conclusions

While fluorescence imaging is a highly efficient method for documenting nearly all steps of the viral life cycle, it is limited by the diffraction limit of light. Advanced resolution fluorescence microscopies have overcome this limit; nevertheless, their effective spatial resolution—particularly in the axial dimension (~50 nm)—is barely sufficient to resolve small HBV structures, including mature Dane particles (~42 nm), capsids (~30 nm), and especially subviral particles (SVPs, ~22 nm). Yet, it remains difficult to track the HBV intracellular pathway by imaging for the following reasons. (i) The compaction of the genome, which includes several overlaps between open reading frames, is not amenable to the insertion of a DNA sequence coding for a reporter protein such as GFP. Despite the advantage of introducing a series of DNA stem loops such as MS2, recognized by the chimeric protein MS2-GFP to track the viral genome, this construction may not be compatible with encapsidation [138]. Furthermore, if sequences are introduced into the Pol domain, it will then be necessary to provide this protein in trans, which has consequences for viral production [139]. Other short sequences (HA or Hibit) have been introduced into the preCore, PreS1, or PreS2 regions, allowing the observation of multiple steps in the viral cycle and opening the possibility of inserting fluorescent reporter fragments to track these replication steps via microscopy in a cellular context [31,40,140]. However, replacing these small tags with large fluorescent proteins remains to be characterized. (ii) The massive production of non-infectious subviral particles in addition to virions also represents a limitation; indeed, HBV virions represent only a minute fraction of SVPs and empty virions. (iii)Although electron microscopy provides nanometer-scale resolution, it only allows the observation of static images. In contrast, nucleic acids are highly dynamic within cells, making it difficult to capture their localization at a specific point in time.

Moreover, even though FISH has allowed the imaging of particle localization by confocal fluorescence and high-resolution microscopy, fixation is required. Techniques permitting live-cell imaging are difficult to implement in combination with FISH imaging techniques. In addition, it is evident that extensive treatment of cells, such as background bleaching, fixation, permeabilization, deproteinization, and NA digestion, might hamper reproducibility in IF-FISH experiments, and this raises the question of whether different experimental setups might give different experimental outcomes in the context of dot-based image analysis. Beyond this, using the combined detection of proteins and NAs in single-cell microscopy analysis will become the new way of understanding the HBV replication cycle.

## Figures and Tables

**Table 1:** Fluorescent probes targeting the various types of NAs produced during HBV replication. Three main PS have been designed to detect HBV RNAs and DNAs. Probe set ~2000–3000 detects pgRNA (and preC mRNA) and (+) DNA depending on experimental conditions (RNAse/DNAse and DNA denaturation) but does not detect subgenomic RNAs. A second probe set, ~3000–800 targets the minus (−) DNA strand (rcDNA) together with replication intermediates, including cccDNA and pf-rcDNA in the nucleus. A third probe set, ~1000–1500, targets the gap region of the (+) strand such cccDNA. Under conditions in which samples are treated RNAse-free DNAse I, this probe set can also detect pgRNA and subgenomic RNAs in the cytoplasm.

Reference	Probe Set (PS)	Genome Position/Template	Target	FISH Technology
[109]	PS 1	nt 1987–2805	pgRNA and spliced RNAs	RNA smFISH
[110,111,112] ^a^	PS 1 PS 2 PS 3	nt 1930–2900 nt 2957–837 nt 500–1590	pgRNA and (+) DNA (−) DNA (+) DNA (gap region)	b-DNA
[113] ^b^	PS 2 PS 3	nt 2959–837 nt 481–1541	(−) DNA (+) DNA	b-DNA
[114] ^c^			CRISPR-Tag (intron region) specific probes	rcccDNA-CRISPR-Tag
[115] ^c^	HBV DHBV	CMV-ayw (HBV) ^b^ pSP65-Gal5.1 (DHBV) ^b^	(−) DNA cccDNA	*In house* DNA fluorescence in situ hybridization (FISH).
[116]		nt 1518–1542	pgRNA (+) DNA	*In house* RNA fluorescence in situ hybridization (FISH).
[117] ^c^		Biotin labeled probes	all viral transcripts	*In house* DNA fluorescence in situ hybridization (FISH)
[118] ^b^	PS 1 PS 2	nt 2959–837 nt 1091–1557	(−) DNA (HBV DNA) (+) DNA (cccDNA, vRNAs)	b-DNA
[119] ^c^		whole genome	(−) DNA	

^a^ The three references [110,111,112] have been grouped together even though the three sets have some different residues. ^b^ The protocol and use of the probes in this cluster of references are summarized in HBV Research protocol https://ice-hbv.org/protocol/a-fluorescent-in-situ-hybridization-fish-assay-for-detection-of-hbv-dna-in-cell-culture-models/, accessed on 1 July 2026. ^c^ Whole genome plasmids were used as probe templates.

## Data Availability

All data is available upon request to VSV or to HdR.

## Abbreviations

NA (s), Nucleic acid (s); PS (s), Probe set (s); HBV, Hepatitis B Virus; Covalently Closed Circular DNA, cccDNA; rcDNA, relaxed circular DNA; pgRNA, pre-genomic RNA; protein-free rcDNA, pf-rcDNA; double-stranded DNA, dsDNA; Stimulated Emission Depletion, STED; Nucleocapsid, NC.
